# Communication in non-communicable diseases (NCDs) and role of immunomodulatory nutraceuticals in their management

**DOI:** 10.3389/fnut.2022.966152

**Published:** 2022-09-21

**Authors:** Abhiram Kumar, Kalyani Sakhare, Dwaipayan Bhattacharya, Raktim Chattopadhyay, Purvish Parikh, Kumar P. Narayan, Anubhab Mukherjee

**Affiliations:** ^1^Esperer Onco Nutrition Pvt. Ltd., Mumbai, India; ^2^Department of Biological Sciences, Birla Institute of Technology and Science – Pilani, Hyderabad, India; ^3^Department of Clinical Haematology, Mahatma Gandhi Medical College and Hospital, Jaipur, India

**Keywords:** gut microbiota, dysbiosis, NCDs, immunomodulatory nutritional intervention, probiotics

## Abstract

Conveyance of pathogens between organisms causes communicable diseases. On the other hand, a non-communicable disease (NCD) was always thought to have no causative transmissible infective agents. Today, this clear distinction is increasingly getting blurred and NCDs are found to be associated with some transmissible components. The human microbiota carries a congregation of microbes, the majority and the most widely studied being bacteria in the gut. The adult human gut harbors ginormous inhabitant microbes, and the microbiome accommodates 150-fold more genes than the host genome. Microbial communities share a mutually beneficial relationship with the host, especially with respect to host physiology including digestion, immune responses, and metabolism. This review delineates the connection between environmental factors such as infections leading to gut dysbiosis and NCDs and explores the evidence regarding possible causal link between them. We also discuss the evidence regarding the value of appropriate therapeutic immunomodulatory nutritional interventions to reduce the development of such diseases. We behold such immunomodulatory effects have the potential to influence in various NCDs and restore homeostasis. We believe that the beginning of the era of microbiota-oriented personalized treatment modalities is not far away.

## Graphical Abstract

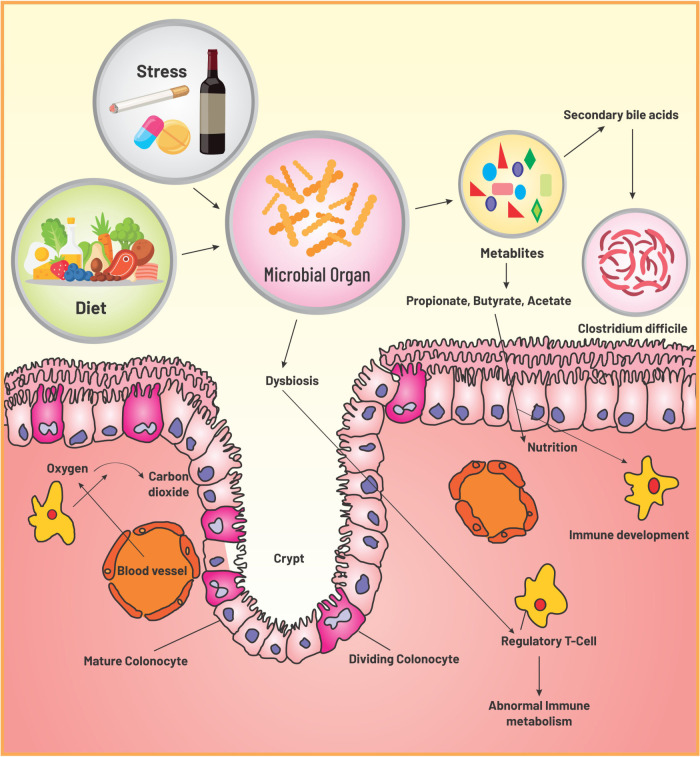

## Introduction

As comprehended since centuries, an infectious (communicable) disease is a malady in which a particular infectious agent (or its toxins) gets transmitted from an individual to a susceptible host. On the other hand, a non-communicable disease (NCD) has no causative agents to be transmitted. As time rolled on, scrupulous scientific observation has stumbled upon an expanding number of NCDs that cognate with a contagious pathogenic risk factor. These findings actually have blurred the distinction between a communicable disease and a non-communicable disease, although there were clear demarcations between them earlier (1, 2). It is now well known that the human microbiota comprises a multitude of microorganisms, the majority and the most widely studied being bacteria residing in the gut. The adult gut harbors almost 100 trillion resident microbes, and the corresponding genome (microbiome) contains 150-fold more genes than the host genome itself. Needless to mention, these complex microbial communities have grown in the evolutionary pathway to maintain a symbiotic relation with the host physiology influencing digestion, immune responses, and metabolism. However, it is not yet absolutely clear to which magnitude microbial spread can contribute to the onset of NCDs rendering it as a subject for intense investigation. Nonetheless, it is assiduously arduous to decouple environment from microbiota, which renders the investigation of the transmissibility of NCDs a bit challenging (3).

Robert Koch’s postulates have been entrenched in microbiologists of a different era as necessary and sufficient conditions to be fulfilled – which states that a specific microorganism engenders specific disease. Though limited to some extent, these postulates can still be considered as a scaffold to decipher microbial causes of ailments (4). Similarly, a way to be able to establish some causal links beyond mere correlations between dysbiotic gut microbiota and occurrence of NCDs is therefore needed (5). For instance, in case of cardiovascular diseases (CVD), cogent evidence is accumulated, which emphasizes a correlation with the CutC enzyme (encoded by gut microbe), known to convert carnitine and phosphocholine compounds to trimethylamine (TMA). Thereafter, it reaches the host liver and gets oxidized into trimethylamine oxide (TMAO), which is proven to exert an impact on cholesterol metabolism and to enhance atherosclerosis development (6). It has become an exigent demand to identify various environmental risk factors (in addition to the genetic risk factors) to establish molecular pathways causally linking them with NCDs such as CVD, diabetes, osteoporosis, polycystic ovary disease (PCOD), and non-alcoholic fatty liver disease (NAFLD). Of many environmental components, microorganisms play crucial roles in pathogenesis and progression of cancer. Recent studies have proven that infectious agents can cause more than 20% of all cancers, such as *Helicobacter pylori* causes gastric cancer, hepatitis B and C virus can trigger the onset of liver cancer, while cervical cancer takes its origin from *human papillomavirus* (7, 8). Besides probing the effects of single contagious microorganism in tumorigenesis, scientists have also undertaken the investigation of tumor environment associated microbial pool which has been described as a crucial environmental factor for some cancers, including colorectum, liver, biliary tract, and breast cancer (9). Intestinal epithelium involves in a cross talk with trillions of bacteria sheltering in the colorectum (10). They have immense importance toward the maintenance of physiology of GI tract in terms of energy balance and immunity. It is obvious that alterations in their ratio of quantity can switch the equipoise, which can eventually result in intestinal and extraintestinal maladies (11, 12).

As ratiocinated from many preclinical and clinical evidences across the globe, most of the NCDs and cancers caused by infections are associated with systemic inflammation. Thus, it is counterintuitive that phytopharmaceuticals having anti-inflammatory properties would be deciphered as potential therapeutic arsenals against these bleak maladies. This review is an attempt to summarize all recent accomplishments of immunomodulatory nutraceuticals in the prevention or management of various NCDs mitigating the ominous effects of pathogenic infection. We also propose that phytonutrients should be accompanied by beneficial commensals (probiotics) while developing therapeutic nutraceutical formula to combat NCDs and some cancers. This will provide nutritional interventions to address the suboptimally treated infections to trigger the immunomodulation to combat the chronic inflammatory state caused by the pathogenic infection with subsequent disease regression. In the following sections, we will elaborately discuss the communicable part in the NCDs, gut dysbiosis, microbial origin of NCDs, and emergence of immunomodulatory nutraceuticals as a therapeutic modality.

## Communicable diseases and non-communicable diseases

**FIGURE 1:**
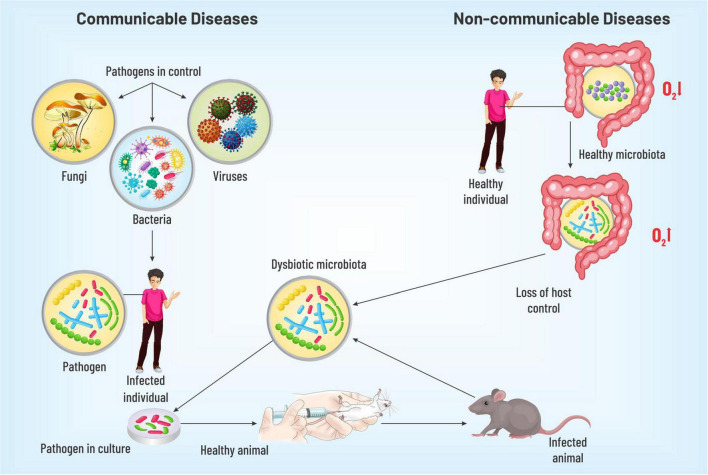
A diagrammatic representation discussing the identification of pathogenesis for communicable and non-communicable diseases. The dysbiotic gut microbiota on injection into the healthy model organism isolated from individuals with NCDs was observed to cause the respective disease thus verifying the hypothesis of gut microbiota playing a key role in disease pathogenesis.

As we touched upon the origin of communicable diseases, various pathogens are transmitted in various ways (dermal, sexual, oral, fecal, respiratory, and bites) between various organisms, which have a tremendous potential to eventually make a multitude sick. To further probe into the details about the origin of the diseases, numerous chronological details can be cited. In 1546, Girolamo Fracastoro proposed that microorganisms might beget human ailments. Yet, it was not accepted for a lack of causal connection. A technology was discovered by Louis Pasteur in 1864 to associate microorganisms with transmissible maladies. This was followed by a demonstration of a microbial link for an illness of silkworms by the same scientist in 1870. Robert Koch proposed a theory in 1892, popularly known as Koch’s postulates, to emphatically establish the causal link between microbes and transmissible ailments, which were used for tuberculosis, the foremost reason of mortality at that time. Recognized as ‘germ theory,’ the postulates can be summarized as (i) the causative species will be present throughout the ailment (ii) which can be isolated and propagated *in vitro*, (iii) the cultured microbe must cause the disease *in vivo*, and (iv) it can be reisolated from the new host exhibiting identical properties as the original. This spawned an array of seminal contributions over the following two decades, understanding microbial origins for cholera, typhoid, diphtheria, tetanus, and bubonic pest and bacillary dysentery with the invention of modalities to treat them (vaccine, antibiotics, etc.). Subsequent scientific movements aptly confirmed ‘germ theory,’ the proposition that a specific microbe can cause a specific transmissible malady. The cornerstone of the concept was “pathogen-control” over the host, meaning thereby pathogens have the potential to defeat host defenses in individuals with an unimpaired immunity (4). Although there is no denying the fact that communicable diseases remain the leading causes of human mortality globally, still, in high-income countries, the foremost health challenges are NCDs, such as CVDs, some cancers, habitual respiratory conditions, diabetes, arthritis, and asthma. NCDs were initially conceived as maladies that are steered by rudimentary flaws in host physiology not transmittable directly from one person to another and are not caused by a contagious agent. Thus, the germ theory was not directly applied to NCDs. Strikingly, research exploration in the last few decades has tied the microbial pool inhabiting the large intestine to numerous NCDs. Therefore, as the germ theory lays down a theoretical framework for transmissible ailments, there has been an urgent exigency to enunciate a speculative scaffold to decipher the balance and imbalance between the host and microbial organ, correlating gut microbes with NCDs. A new terminology - ‘germ-organ theory’ is proposed to delineate the ideation. Unlike the ‘germ theory’ that portrays the pathogen in control, the cornerstone of the ‘germ-organ theory’ is the host-control over the microbial ecosystem. In order to build a theoretical overarching – Koch’s postulates need to be modified for the microbial origin of the NCDs, where the causative pathogenic invader would be replaced by a dysbiotic microbiota (DM). Thus, modified postulates will read like (i) DM is found in people having NCDs, (ii) which can be isolated and propagated in *in vitro* culture, (iii) it can originate the disease when transferred to a healthy host *in vivo*, and (iv) it can be reisolated from the new host. This set of principles can largely be applied to various NCDs such as CVDs, T2DM, IBD, and their risk factors such as obesity. In the following section, we shall map the connection between dysbiotic microbiota, host immune system, and NCDs (4, 13–17) (Figure 1).

## Gut microbiota: Nutrition, immunity, and dysbiosis

**FIGURE 2:**
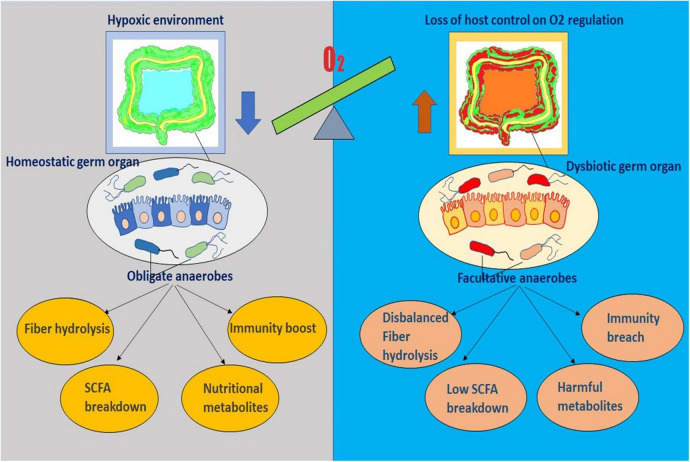
The diagram represents the sensitive interplay existing between the gut microenvironment and the residing microflora. It outlines the key role of oxygen imbalance in dysbiosis and diseased physiology.

Unlike invertebrates where a remarkable host control is manifested due to bacteria-specific peptides, diversified microbiota in mammalian gut does not advocate for species selection. Mammals control the composition of infant gut via milk oligosaccharides and of adult gut by secretion of antimicrobial proteins (e.g., defensins) (18–20). With growing knowledge, it is now evident that in order to maintain gut homeostasis, which is critically dependent on predominance of obligate anaerobes of *Bacteroidetes phylum* and Clostridia class, host limits the oxygen supply in the large intestine by keeping the colonic epithelium in hypoxia (21). This induces self-assembly of the coexisting microbial community with the formation of an anaerobic trophic network where every single location stands in for a nutrient niche to be occupied by a particular microbe having necessary metabolic activities creating similar metabolic patterns in individuals (22–24). Gut homeostasis, a stable equilibration maintained between host immunity and microbiota, is associated with hydrolysis and fermentation of complex structures of fibers by the obligate anaerobes into a bountiful of short-chain fatty acids (SCFA) metabolites – butyrate, propionate, acetate, and plentiful of other metabolites, where most of the SCFAs are assimilated by the host for its nutrition (25, 26). Thus, host-driven anaerobiosis inhibits the nutritional competition of the obligate anaerobes with the host for fiber metabolites, inhibits further catabolism of the metabolites to CO_2_ by facultative anaerobes, and steers its control over the microbial community without inflicting restriction on the composition. Moreover, these SCFA metabolites essentially are instrumental in immune development in various ways such as via inhibiting dendritic cell differentiation, promoting colonic and peripheral Treg production, and modulating function of intestinal macrophages. Importantly, homeostasis also requires a perpetual flow of SCFAs in epithelial cells of the colon to fuel PPARγ, the intracellular sensor for butyrate, and to stimulate Treg proliferation in colonic mucosa (27–29). These two mechanisms together propel the metabolism process in the direction of mitochondrial β-oxidation, which demands an ample amount of oxygen, and induce hypoxia in colonic epithelial cells (21, 30). To summarize epithelial hypoxia guarantees SCFA production by obligate anaerobes and SCFAs engender colonic epithelial hypoxia – thus, participating in a virtuous cycle that conserves gut homeostasis and preserves microbial ecosystem (Figure 2).

In general, two major resident phyla in adult gut are Gram-negative *Bacteroidetes* and Gram-positive *Firmicutes*, with a lower abundance of actinobacteria, cyanobacteria, fusobacteria, proteobacteria, and verrucomicrobia (31). Lipopolysaccharides (LPS) present in the cell walls of Gram-negative strains trigger a strong inflammatory response in the host in order to defend against infectious invaders. As we discussed above, normalcy in tissue is maintained by well-orchestrated regulatory responses by impeding excess inflammation. It is worth mentioning here that the matrix of exposure to microbes in early life is of immense importance to build up a resilient immune regulation for the host and disruption of either host immune response or microbiota can induce chronic inflammation (23, 32–34). Unlike conventional comprehension of such as acute inflammation, chronic inflammation associated with obesity, atherosclerosis, diabetes, allergy, and asthma represents a chronic dysfunction of the tissue and a metabolic shift from homeostasis to an imbalanced state (35).

Gut dysbiosis can be stated as a disorder in the microbial organ as the host loses control over it. Microbial dysfunction causes imbalances in metabolite production affecting multiple organs and leading to various NCDs (26, 36, 37). This disruption incapacitates the host curbing the stream of oxygen into the colon – leading to a major switch in the microbial signature – a dominance of facultative over obligate anaerobes (38, 39). Augmentation of the facultative anaerobe (*phylum proteobacteria*) is observed in humans inclined to a western-style diet or receiving antibiotic therapy or suffering from IBS, IBD, metabolic syndrome, necrotizing enterocolitis, or colorectal cancer and also in various animal models of colitis and colorectal cancer (19). It is well known that antibiotics deplete the gut microbes lowering the concentration of SCFAs, which are pivotal for homeostasis. In addition, enteric facultative pathogens cause intestinal inflammation promoting transepithelial migration of neutrophils, which devour Clostridia resulting in a diminution in SCFAs (39, 40). Both the mechanisms corroborate a switch in energy metabolism from mitochondrial β-oxidation to anaerobic glycolysis, the latter escalates the oxygen levels exuding from the host colonic epithelium. Besides depletion of SCFAs, both pseudomembrane formation and colonic crypt hyperplasia contribute to an accretion of Proteobacteria via boosting the level of oxygen (and/or other respiratory electron acceptor) in the large intestine. In the following section, we will elaborately discuss how gut dysbiosis can lead to the occurrence of various NCDs.

## Gut microbiome and various non-communicable diseases

**FIGURE 3:**
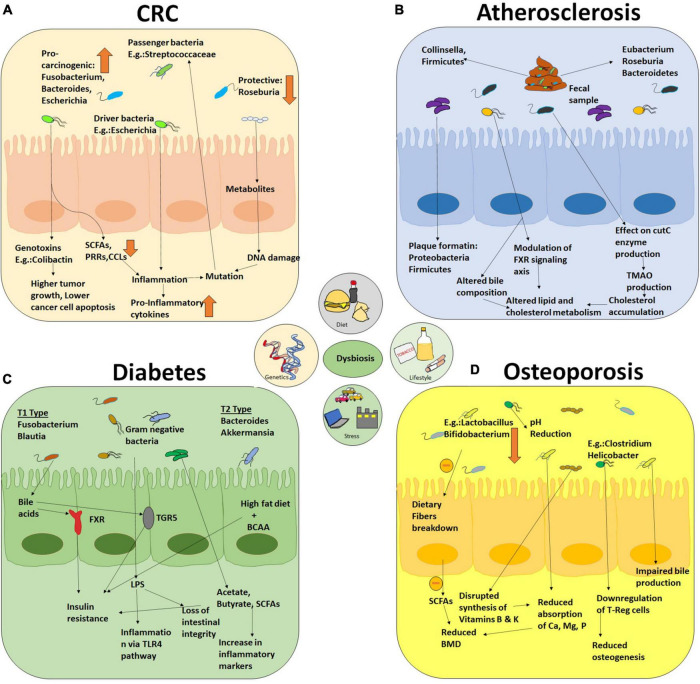
An overall holistic view of the workings of dysbiotic microbiota that contribute to disease pathophysiology of NCDs and some cancers. The figure includes four major diseases **(A)** colorectal cancer, **(B)** atherosclerosis, **(C)** diabetes, and **(D)** osteoporosis as examples of the detrimental effects produced by dysbiosis in diseased individuals.

As we have already started gathering, depending upon modulation of physiological, environmental, genetic, and behavioral factors for a prolonged time, various NCD conditions arise. Each year, 41 million human demises are caused by NCDs, and this death rate is 71% worldwide. More than 15 million individuals die from NCDs every year, and their ages are between 30 and 69 years. Almost 77% of all NCD deaths occur in low- and middle-income countries only. Below, we will attempt to create some causal links, beyond mere correlation, between alterations in gut microbial ecology with the onset of NCDs (3, 41). Table 1 summarizes the causative roles of microorganisms in the pathogenesis of various NCDs. Figure 3 depicts the detrimental effects produced by dysbiosis in diseased individuals in colorectal cancer, atherosclerosis, diabetes, and osteoporosis.

**TABLE 1:** Summary of causative microorganisms contributing to the pathophysiology of various NCDs and colorectal cancer.

Diseases	Cause	Mechanism	References
Colorectal cancer	*Fusobacterium nucleatum*	Enhanced tumorigenesis by inducing interleukin production, p-STAT3, p-STAT5, and p-ERK.	(102)
	*Enterotoxigenic Bacteroides fragilis (ETBF)*	Promoted CRC development by inducing pro-inflammatory cytokines, targeting Wnt signaling.	(103)
	*Enterococcus faecalis*	Enhanced the CRC development through Wnt/B-catenin signaling, activating the transcription factors functioning in de-differentiation.	(104)
	*Escherichia coli*	Supported tumorigenesis by overexpressing virulence genes encoding effectors and toxins such as cycle inhibiting factors, cytotoxic neutralizing factors, cytolethal distending toxins, and colibactin thereby inducing carcinogenesis.	(105)
	Species of *Clostridium, Bacteroides, Escherichia*, and *Enterococcus* genera	Played a role in development of CRC by increasing crypts upon induction by 1,2-dimethylhydrazine	(45)
Atherosclerosis	*Chlamydia pneumonia*	Promoted foam cell formation, recruit leukocytes, proliferation of smooth muscles, and lesion progression by infecting macrophages.	(106)
	*Porphyromonas gingivalis*	Promoted low-grade inflammation	(107)
	Proteobacteria (*Chryseomonas* and *Helicobacter*) Actinobacteria (*Collinsella*)	Activated inflammatory pathways through Toll-like receptors (TLRs) on activation with LPS.	(108)
Diabetes	*Fusobacterium nucleatum* and *Ruminococcus gnavus*	Increase pro-inflammatory cytokines along with their role in development of other diseases.	(109)
	*Prevotella copri* and *Bacteroides vulgatus*	High-fat diet enriched with BCAA promotes insulin resistance and increases the risk for T2D development.	(73)
Osteoporosis	Reduced *Clostridium* sp., increased *E. coli*	Estrogen deficiency reduced microbial diversity thereby destroying immune homeostasis. Altered nutrient absorption.	(118)
Polycystic ovarian syndrome	Higher *Porphyromonas* spp., *Bacteroides coprophilus*, *Blautia* spp., and *Faecalibacterium prausnitzi*i, and lower *Anaerococcus* spp., *Roseburia* spp., *Odoribacter* spp., and *Ruminococcus bromii*	Increased gut permeability, increased endoxemia, activated immune system, hyperinsulinemia, increasing the production of ovarian androgen	(111)
	Gut microbial metabolites such as increased LPS (Gram-negative bacteria), increased GLP-1 (*Bifidobacterium* spp.). Decreased levels of Glycodeoxycholic acid (GDCA) and tauroursodeoxycholic acid (TUDCA) (*Bacteroides forsythus*)	Increased LPS attaching to CD14/TLR4 in macrophages induced the secretion of pro-inflammatory cytokines participating in insulin resistance and diabetes. Increased GLP-1 affected GI system and CNS via vagus nerve. Decreased GDCA and TUDCA uncoupled bile acids synthesis in PCOS patients	(112, 113)
Non-alcoholic fatty liver disease	*Bacteroides* sp.	Decreased levels of SCFAs and amino acids	(114)
	*Saccharomyces cerevisiae, Lactobacillus fermentum, Weissella confuse, S. cerevisiae*, and *W. confusa*	Increased levels of ethanol leading to progression of NASH through oxidative stress and liver inflammation.	(115)
Obesity	*Enterobacteriaceae* and *Desulfovibrionaceae*	Contains LPS as an endotoxin that strongly caused inflammation on entering the blood system.	(116)
	*Enterobacter cloacae* str. B29	Presence of this microorganism in the gut promoted the induction of pro-inflammatory cytokines due to increased endotoxin levels eventually resulting in insulin resistance and accumulation of fat because of dysregulated lipid metabolism when supplemented with high-fat diet.	(96)
Aging	Reduced ratio of *Bacteroides/Firmicutes (Firmicutes, Bacteroidetes, and Proteobacteria*, which can reach 70%, 30%, and 5% of the total abundance). Prevalence of pro-inflammatory *enterobacteria, streptococci, staphylococci, fusobacteria*	Decreased immune system function, increased inflammatory state.	(117)

### Colorectal cancer

In 2018, colorectal cancer is the standard type of cancer ranked second in mortality and third in incidence globally (42). Approximately 70% of the human microbiome resides inside the colon (43). The genetic heritability of CRC is as low as 10–12%, which explains the role of microenvironment in the development of sporadic CRC (44). Several studies indicated the next part of gut microbes in CRC development. Microbial species such as *Clostridium*, *Bacteroides*, *Enterococcus*, and *Escherichia* genera were found to induce carcinogenesis by 1,2-dimethylhydrazine (45). A fecal transplant further established the role of gut microbiota in colorectum from patients with CRC to germ-free mice, which developed tumors when supplemented with azoxymethane to induce colon neoplasia (46). The studies showed the abundance of procarcinogens microbes belonging to *Fusobacterium*, *Bacteroides*, *Porphyromonas*, and *Escherichia* class with declined protective microbes such as *Roseburia* in patients with CRC (47–49).

The gut microbiome impacts on the development of CRC via different mechanisms, which include the microbial factors (genotoxins or metabolites). The drivers in this mechanistic pathway are SCFAs, PRRs, and several chemotactic factors (CCL17, CCL20, CXCL9, and CXCL10). Cytolethal distending toxin (CDT) and colibactin are commonly known toxins produced by *Escherichia*, *Campylobacter*, and *Enterobacteriaceae* spp. Some microbes may act as carcinogenic (driver microbes) and some as opportunistic bacteria (passenger bacteria) in the tumor microenvironment. This host microorganism interaction leads to the activation of several downstream signaling pathways, resulting in CRC development (50).

To describe the polymicrobial interaction in the tumor environment of CRC, Tjalsma et al. put forward the driver–passenger model. This model proposed that the driver bacterium such as *Bacteroides fragilis* and *Escherichia coli* in the tumor microenvironment releases genotoxins that promote inflammation causing premature transformations in adenocarcinoma cells thereby causing a favorable tumor microenvironment for the growth of passenger microorganisms. Passenger bacterium such as *Streptococcaceae* and *Coriobacteriaceae* proliferate in the tumor microenvironment in such a way that can outgrow the other bacterial species. The development of microbial biomarkers depends on different tumor stages. Identification of various bacterial strains surviving across different carcinogenic stages is also important in this aspect. While the driver bacteria can be directed against colorectal cancer, the passenger bacteria is associated with disease initiation, propagation, and response to treatment (51).

Ingestion of non-digestible carbohydrates leads to the production of SCFAs due to microbial fermentation in the colon. Butyrate is known as an anti-inflammatory and tumor-suppressive entity that induces apoptosis in CRC. Reduced levels of SCFAs are linked with a high risk of CRC, advanced colorectal adenoma, and ulcerative colitis development (52–54). For screening CRC, accurate biomarkers are needed, which if detected at early stages, can be treated with clinical therapies. The fecal immunochemical test (FIT) is currently used to detect CRC, with the significant drawback being 79% sensitive and 25–27% susceptible to detect advanced colorectal adenomas. Several studies have reported the utilization of microbial species to detect CRC using butyryl-Co-A dehydrogenase from *F. nucleatum* and RNA polymerase B subunit from *P. micra* quantified by PCR (49). Several reports established the association between oral microflora such as *Prevotella* and *Streptococcus* and CRC opening a new array for predicting CRC.

### Atherosclerosis

In recent years, a new dimension of understanding has emerged on the pathogenesis of cardiovascular diseases, beyond the interaction of nutrition and genetic variation. Preclinical and clinical evidences have revealed that gut microbiota influences the pathogenesis of atherosclerosis (55). An involuted indication involves both inflammatory and metabolic pathways which are leveraged by the gut microbiota in three pathways: (i) acceleration of plaque formation via turning on the inflammatory immune response caused by local or distant infection, (ii) generation of ominous bacterial metabolites (viz., TMAO) from specific components of diet, and (iii) disruption of cholesterol and lipid metabolism routes of the host (56). As evident from the scientific studies discovering the presence of bacterial DNA in plaques, both direct and distant infections contribute to atherosclerotic plaques development. These bacteria are also found mostly in gut and oral cavity (57). To our intrigue, 16S rRNA analysis revealed a large abundance of *Firmicutes* and *Proteobacteria phyla* in the atherosclerotic plaques (58). Besides these, pathogenic *Helicobacteraceae* and *Neisseriaceae* were also found to be more prevalent in patients with symptomatic atherosclerosis (59). 16S shotgun sequencing experiment of fecal samples from healthy individuals, patients with atherosclerosis, and healthy volunteers with symptomatic atherosclerosis was found to vary in various aspects. Patients with disease symptoms were reported an enhancement in genus *Collinsella*, whereas *Eubacterium* and *Roseburia* were increased in controls. Moreover, gut dysbiosis in the patients was accompanied by an augmentation of an abundance of inflammatory genes (60). The abundance of *Firmicutes* got increased and *Bacteroidetes* got decreased when compared with healthy volunteer (61). Several preclinical and clinical studies have suggested strong causal links between gut microbiome and atherosclerosis. Compelling evidence is accumulated, which emphasizes a correlation with the CutC enzyme (encoded by gut microbe), known to convert carnitine and phosphocholine compounds to trimethylamine (TMA). Thereafter, it reaches the host liver and gets oxidized to trimethylamine oxide (TMAO), which was proven to affect cholesterol metabolism-enhancing cholesterol accumulation in macrophages and atherosclerosis development (6).

It is also evident from a few studies that patients with atherosclerosis have modified lipid metabolism, and plasma cholesterol levels are correlated with bacteria in the gut and oral cavity (57). Mechanistically, gut microbiota modulates cholesterol and lipid metabolism through altering bile acids and its farnesoid X nuclear receptor (FXR). It is now understood that bile acids help in the absorption of dietary lipids and fat-soluble vitamins. In the liver, primary bile acids are generated by being conjugated to taurine or glycine, formation of secondary bile acids is achieved by intestinal decoupling by bacterial bile salt hydrolase followed by colonic metabolism. However, gut microbial modulation of bile acid composition and signaling in FXR-expressing tissues in atherosclerosis is not been fully explored yet and remains as a matter of intense investigation (62). There is no denying the fact that a perpetual interaction between host and gut microbiota over a life span would determine the speed of disease propagation. To establish the causal relationship between a species or metabolites and atherosclerosis, it is, therefore, imperative to establish proper linkages of microbiota to distinct functions.

### Diabetes

The widespread presence of obesity in the majority of the population increases the development of other metabolic NCDs such as CVD and T2DM (63). These disorders alone contribute to approximately 80% of premature mortality, with diabetes mellitus affecting nearly 347 million people globally (64). Lifestyle, obesity, malnutrition, and other health-related disorders contribute majorly to CVD and T2D in the Indian population (65). Recent studies showed the link between diet and the microbiome inhabiting the gut and their contribution to food digestion and absorption. Studies reported that the *Bifidobacteria, Prevotella*, and *Akkermansia* majorly inhabit individuals consuming the plant-based fiber-rich diet. In T2DM, *Bacteroides*, *Akkermansia, Ruminococcus*, and *Faecalibacterium* were found in abundance, while *Roseburia* were found to be in lower concentrations. Intestinal microbiota, disruption in the intestinal barrier, and inflammation affect the development of diabetes and obesity. Alteration in gut microflora due to external factors such as diet affects the metabolism causing diabetes and insulin resistance. LPS secreted by Gram-negative gut microflora produces inflammatory cytokines causing inflammation through TLR4 signaling pathway (66). The increased LPS was also found to destroy the integrity of the intestine, causing high LPS absorption. While acetic acid and butyrate have been reported to enhance the intestinal barrier functioning linked to insulin resistance and inflammation, SCFAs are also known to regulate glucose homeostasis through G protein activation of the Langerhans cells (67, 68).

When fecal bacteria transplantation was performed in insulin-resistant patients from insulin-sensitive patients, significant insulin sensitivity was observed with an abundance of butyrate-producing bacteria, which was observed on analysis as *Faecalibacterium prausnitzii* (69). Gut microbes transform the bile acid synthesized in the liver into secondary bile acids through enzyme metabolism. These secondary bile acids regulate insulin sensitivity through Farnesoid X receptor (FXR) and the Takeda G protein-coupled receptor 5 (TGR5) (70). Branched-chain amino acids (BCAA) are a critical predictive marker for T2D and are associated with the risk of developing T2D (71, 72).

A high-fat diet with BCAA also leads to insulin resistance has also been reported. Other studies with humans supported that BCAA with a high fiber supplementation escalates the risk of T2D development. The microflora associated with BCAA synthesis is majorly *Prevotella copri* and *Bacteroides vulgatus* (73).

### Osteoporosis

Osteoporosis is a disease often marked by decreased bone mass per unit volume and wear and tear of bone tissue microstructure, increasing the susceptibility to fracture. It can occur irrespective of age and sex and is common in postmenopausal women. Nutrition, heredity, lifestyle, and hormone levels are known to contribute toward the development of the malady. The four distinct phases of the bone remodeling cycle comprise initiation, resorption, reversal, and formation. Endosteal cells, osteoclasts, osteoblasts, and osteocytes together form the bone remodeling units that participate in the bone renewal process (74). The main regulators participating in bone metabolism include vitamin D, estrogen, inflammatory factors, and parathyroid hormones. Menopausal women often manifest primary osteoporosis thereby rendering menopause as a major risk factor of the same (75). In contrast, pathological factors such as inflammatory bowel disease, parathyroid disease, glucocorticoid therapy, type 1 diabetes, arthritis, and smoking lead to secondary osteoporosis’s onset (76).

The gut microbial composition can surely influence nutrient absorption. Upraised abundances of microbes such as *lactobacillus* and *bifidobacteria* were shown to inhabit the human intestinal tract, promoting the absorption of phosphorous, calcium, and magnesium, leading to increased bone mineral density (BMD) (77). It was also shown that the diversity of gut microbes could affect the pH of the gut, further affecting nutrient absorption, specifically, calcium absorption. These microbes also participate in bile acid metabolism and vitamin B and K synthesis, playing a crucial role in bone health and calcium absorption. The gut microbiota catabolizes the macromolecules into simpler components facilitating the ease of nutrient absorption by increasing the bone density and delaying the onset of osteoporosis (78).

Moreover, SCFAs produced by the microbial fermentation of dietary fibers regulate bone mass and osteocyte formation. Bone loss was prevented and bone mass was increased in mice fed with SCFA with ameliorated osteoporotic condition. The importance of a balanced diet in the maintenance of bone health was proved when the fecal transplantation was performed from a high-fat diet mouse to a healthy mouse that adopted the gut microflora, causing osteoporosis in healthy mice. These studies showed that the high-fat diet impairs the micro-environment of bone marrow, causing the poor reorganization of hematopoietic stem cells. HFD was also found to activate PPARg2, thereby enhancing bone marrow lipogenesis and impairing osteoblast formation (79).

According to a few recent studies, a commonality related to immune component is shared between osteoporosis and inflammatory joint disease. Function of gut microbiota may be regulated by Th17/T-reg cells. Several gut bacterial species such as *Clostridium*, *Bifidobacterium*, *Helicobacter*, *Bacteroides*, and *Lactobacillus* facilitate the generation of Treg cells in the colon. SCFAs, vitamin A, and estrogen are key players in maintaining the dynamic balance between Th17/T-reg cells inhibiting osteogenesis (80).

### Polycystic ovarian syndrome

Polycystic ovarian syndrome (PCOS) is an endocrino-pathological disorder in which the ovaries produce exceptionally abnormal levels of the male hormone androgen. With hyperandrogenism, insulin resistance, menstrual disorders, hirsutism, infertility, and anovulation, the principal facets of the syndrome, PCOS is prevalent in 5–7% of women population in their reproductive age (81). PCOS is also accompanied by disorders such as hypertension, endometrial carcinoma, type 2 diabetes mellitus, glucose intolerance, dyslipidemia, insulin resistance, dysfunctional bleeding, cardiovascular disease, and pregnancy loss in the majority of the patients (82). Neuroendocrine, environmental factors, metabolic and immune dysfunctions, and genetics play crucial roles in developing PCOS. While polycystic appearing ovary (PAO) is preclinical, it does not fall under PCOS. In contrast, factors such as insulin resistance, obesity, dopaminergic dysregulation, and stress make the transition of PAO to PCOS in women (83).

**FIGURE 4:**
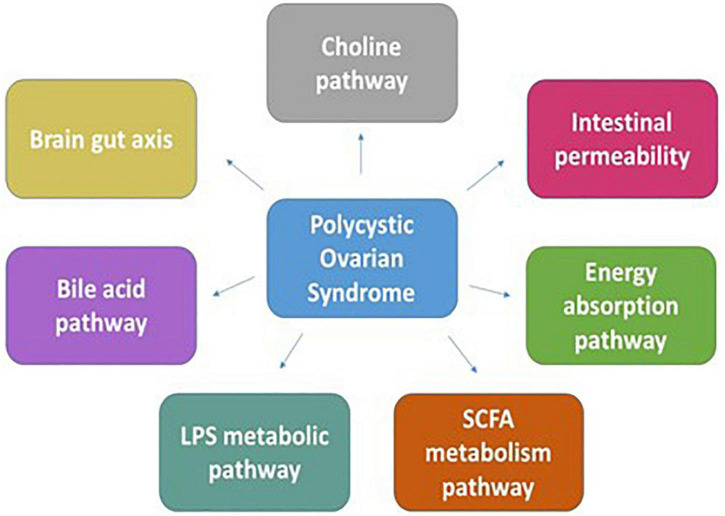
The figure discusses the mechanisms pertaining to the development of PCOS through the cross talk between the genes participating in pathways such as energy absorption, carbohydrate-metabolizing pathways, bile acid pathway, SCFA, and LPS metabolic pathways with the dysbiotic gut microbiota.

Studies showed that the diversity of gut microbes in patients with PCOS is significantly reduced compared with control groups (84). Several genes participating in PCOS development are found to play a role in carbohydrate metabolism and steroid synthesis pathway, thus creating a link between metabolic factors and possible mechanism of PCOS development (85). The occurrence and progress of metabolic and endocrine imbalances in PCOS are affected by the intestinal microflora. In a pilot, clinical study that compared the microbiome from patients with PCOS to control patients, a decrease in *Tenericutes* bacterium (ML615 and S247) was observed (86). It was shown that the gut microbiota disorder in obese patients increases the energy intake of the host as the SCFAs produced from the glucose conversion stimulate the peptide YY release, which prohibits intestinal peristalsis, diminishes the pancreatic discharge, and stimulates energy absorption in the intestine (87, 88). Turnbaugh et al. showed that the Firmicutes population in the intestine was higher, and *Bacteroides* was lower in obese mice that increased the intake and absorption of energy and exhibited the symptoms of obesity (11). Butyrate, acetate, and propionate are the major SCFAs that activate peroxisome proliferator-activated receptor gamma (PPAR-γ), which regulates fatty acid and glucose uptake. SCFAs in the intestines, skeletal muscles, fat, immune system, nervous system, and liver are found to impede appetite-stimulating hormone (ASH) secreted by gastric mucosa that is known to inhibit the secretion of GnRH and other sex hormones. ASH is known to inhibit the CYP19A1 expression that inhibits the conversion of androgen to estrogen. Lower ASH correlates with enhanced androgen levels causing hyperandrogenemia (89). Figure 4 highlights various mechanisms responsible for PCOS development.

### Non-alcoholic fatty liver disease

Non-alcoholic fatty liver disease (NAFLD) is the most common liver disease observed in the past few years. It is indicated by hepatic steatosis which may advance to liver cirrhosis, hepatocellular carcinoma, and non-alcoholic steatohepatitis. Obesity and type 2 diabetes are the major factors that lead to NAFLD development (90). It has been shown that gut dysbiosis correlates with NAFLD. The composition of the gut microbiome differs from NASH, cirrhosis, and fibrosis. The intestinal barrier is compromised due to nutrition stress in NAFLD, which leads to translocating of the gut microbe and their metabolites into the blood system causing hepatic inflammation and cirrhosis (91).

### Obesity

WHO elucidated obesity as unrestrained body fat accumulation leading to increased body mass index (BMI) often caused by an imbalance between energy consumed and expended, measured in terms of calories. In general, BMI greater than 25 is considered as overweight and beyond 30 is known as obese (92, 93). A recent survey by National Health and Nutrition Examination (NHANES) found that more than two in three adults were overweight or had obesity (94). It is on a dramatic rise in low- and middle-income countries, mostly prevalent in urban areas. Nonetheless, obesity is a global burden for our health system and a leading cause of the development of metabolic diseases such as diabetes, atherosclerosis, osteoarthritis, gout, high blood pressure, and some cancers. The shifts in gut microbial composition have been found to play an important role in obesity (95). In 2016, Cindy D. Davis et al. demonstrated that obesity can be attributed to alterations in the composition of gut microbes such as diminished microbial diversity or mutations in bacterial genes and associated metabolic pathways. Obese mice exhibited a higher percentage of *Firmicutes* and half the amount of *Bacteroidetes*, with concurrent augmentation in microbial genes entailing polysaccharide breakdown compared to thin siblings (95).

### Aging-related non-communicable diseases

The gut microbiome provides several benefits such as aiding in digestion and food absorption, metabolization of fibers to produce beneficial SCFAs, nutrient and vitamin synthesis, regulation of host immunity, and integrity of intestine. The gut microbial composition depends on the acidity of the small intestine, stomach and colon, lifestyle, and racial and geographical differences. The microbial composition shifts its function from beneficial to the host toward causing inflammation in individuals with morbid obesity. A similar trend was observed during aging that caused diseases such as CVD, Alzheimer’s disease, insulin resistance, T2DM, and frailty. Compositional differences between these gutbacterial species were observed more in aged population than young individuals. The reduction in gut microbiome diversity was observed concerning age in aged mice, having reduced bacterial biosynthesis of biotin and cobalamin, enhanced creatine degradation and SOS genes associated with DNA repair.

A model, hypothesized to examine the impact of the gut microbiome on aging, was later proved to be correct. It states that microbial dysbiosis increases the intestinal permeability triggering the inflammatory response of the host. This induction of inflammation affects the microbial composition, further promoting dysbiosis creating a feed-forward loop (96). Gut microbial population and its density have influenced systemic inflammation (97). Extrinsic elements including diet and lifestyle determine microbial composition and related dysbiosis (98). When tested on model organisms, the high-fat and meat-dominated diet correlate with microbial dysbiosis (99). In contrast, the Mediterranean diet was shown to induce a healthy gut microbiome (100).

Many gut microbiome and biomolecules synthesized by them, such as SCFAs, vitamins, gut-derived hormones, and other chemical compounds, affect life span and health (101). Table 1 mentions the microbes that modulates the life span.

## Immunomodulatory nutraceuticals toward management of non-communicable diseases

**FIGURE 5:**
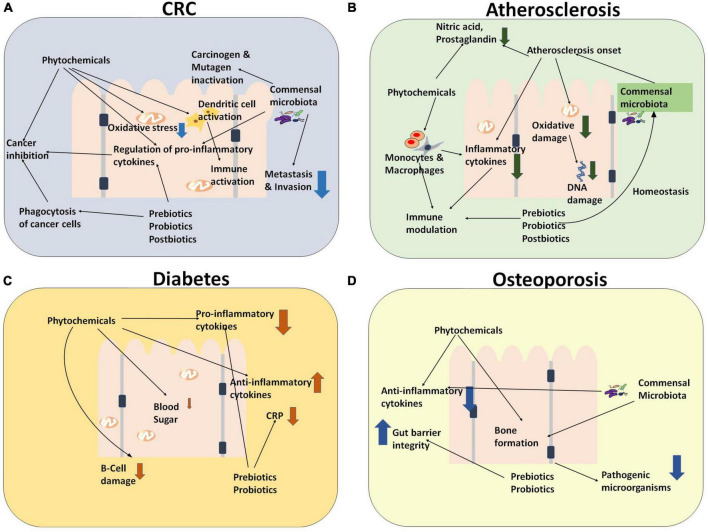
Diagrammatic representation for the immunomodulatory role of various nutraceuticals (such as prebiotics, probiotics, and phytochemicals) wherein they exert a combinatorial effect through several pathways toward the management of **(A)** CRC and NCDs [such as **(B)** Atherosclerosis, **(C)** diabetes, and **(D)** osteoporosis].

It is discernible from our prior discussion that nutraceuticals can play various roles toward the prevention and management of the NCDs. In this section, we shall focus on their immunomodulatory aspects with a view to develop more efficacious formulations. Figure 5 depicts the therapeutic immunomodulatory role of various nutraceuticals (such as prebiotics, probiotics, and phytochemicals) toward the management of CRC, atherosclerosis, diabetes, and osteoporosis.

### Colorectal cancer

Colorectal cancer (CRC) still remains an arduous burden for our health system (118). In 2020, 935,000 deaths have been reported by WHO in both men and women. American Cancer Society (ACS) anticipated that number of new patients with CRC would reach around 2.4 million in the world until the year 2035 (119, 120). Global research would not deny that infection and dysbiosis are closely related to CRC and some nutraceutical compounds including phytochemicals, probiotics, prebiotics, and postbiotics have displayed prominent immunomodulatory roles in the prevention and management of the same. For instance, quercetin, silymarin, and curcumin have shown promising immunomodulatory effects against CRC management, viz., quercetin activated the dendritic cell and thereby antigen presentation, curcumin induced apoptosis and silymarin inhibited Wnt signaling in human colorectal cancer cells (121–124). Few research groups also found some phytonutrient and plant secondary metabolites which exhibited positive outcome toward growth inhibition of CRC by regulating pro-inflammatory cytokines (125, 126). It has been well understood that healthy commensals in gut microbiota such as *L. casei*, *L. plantarum*, *L. bulgaricus*, *L. acidophilus*, and *Bifidobacterium longum* can inactivate carcinogens or mutagens, alter cell differentiation and induce immunomodulatory effects toward growth inhibition of cancer cells (127). Several preclinical and clinical studies demonstrated that prebiotics, probiotics, and postbiotics can improve the immune response such as stimulation of cytokines, viz., IL-6, IL-17, and IL-23, and the downregulation of induction of pro-inflammatory Th17 cells (128). Recently, Yan Li et al. showed that the expression of GPR109A was increased and tumor counts were decreased when postbiotic butyrate got elevated in colon polyposis-bearing mice treated with prebiotic (129). The detailed effect of immunomodulatory nutraceuticals on CRC has been depicted in Figure 5 and Table 2.

**TABLE 2:** Immunomodulatory roles of the nutraceuticals in CRC, and in various NCDs including atherosclerosis, diabetes, osteoporosis, polycystic ovarian syndrome, non-alcoholic fatty liver diseases, obesity, and aging.

Diseases	Nutraceuticals	Source	Bioactivity	References
Colorectal cancer (CRC)	Quercetin	Plant flavanol	Decreased pro-inflammatory cytokines/chemokines production. Activated CD4+ T cells via suppression of mTOR signaling. Induced apoptosis in colon 26, SW480 cells lines.	(198)
	Curcumin	*Curcuma longa*	Decreased expression of cytokines like TNF-α, NF-κB, BCL-2, and epigenetic mutations and subsequently increased the intestinal flora. Inhibited cell proliferation by arresting cells at the G2/M phase.	(199)
	Ashwagandha	*Withania somnifera*	Macrophage-stimulated NO production via NF-κB activation. It significantly influenced amount of leucocytes, neutrophils, lymphocytes and immunoglobulins (Ig) A, M, and G.	(200)
	EGCG	Green tea	IL-17A, IL-8, and HBD-2 expression was markedly increased	(201)
	Allicin	*Allium sativum*	Increased CD4+ T cell, CD8+ T cell, NK cell, and serum IFN-γ. Showed modulation of Nrf2, induced apoptosis, and increased the expression of Bcl-2 and release of cytochrome c.	(202, 203)
	Ellagic acid	Pomegranate extract	Decreased IL-1β, TNF-α, IL-6, IL-17, and IFN-γ. It also induced apoptosis in LNCaP by increasing Bax/Bcl-2 ratio and caspase 3 activations.	(204)
	Apigenin	Parsley extract	Normalized the expression of some colonic inflammatory markers like TNF-α, transformed growth factor-β, IL-6, intercellular adhesion molecule 1 or chemokine (C-C motif) ligand 2	(205)
	Anthocyanidins	Grape seed extract	Prohibited pro-inflammatory NF-κB and COX-2 pathways and prevented cell proliferation via decreasing the nuclear translocation of β-catenin. Anthocyanins showed regulation of gut microbial dysbiosis, reduced the production of ROS in macrophages.	(206)
	Probiotics	*Lactobacillus rhamnosus, Lactobacillus acidophilus Lactobacillus plantarum*	*L. rhamnosus* activates colonic CD8+T to reduce CRC burden *L. acidophilus* decreased cellular proliferation and carcinogenesis. Stimulated the secretion of anti-inflammatory cytokines and upregulation of Treg and Th2 response-related gene expression. *L. plantarum* reduced the tumor growth and increased PD-L1 blocking antibody against cancers.	(207–209)
	Prebiotics	Inulin, FOS	Decreased tumor growth, pointing to an essential role for CD4+ and CD8+ T cells in the inulin-promoted antitumor phenotype.	(210)
	Postbiotics	SCFAs MCT	Lactobacillus Plantarum I-UL4 is a metabolite from probiotics that showed modulation of immune responses *in vitro* and *in vivo*.	(211)
Atherosclerosis	Curcumin	*Curcuma longa*	Reduced pro-inflammatory cytokines in primary human monocytes and increased an anti-inflammatory M2 macrophage phenotype *in vitro*. Decreased atherosclerotic lesion in ApoE and Ldlr double-knockout mice.	(211, 212)
	Arjuna	*Terminalia arjuna* (saponins, tannins, glycosides, and phenolic compounds)	Increased the production of antibodies and delayed-type hypersensitivity using Sheep red blood cells (SRBCs). Stimulated IL-2 and interferon-γ levels but reduced the production of IL-4 in Balb C mice.	(135, 136)
	Allicin	*Allium sativum* L.	Inhibited the production of NO, prostaglandin, and expression of TNF-α, IL-1b, and IL-6 in LPS-activated macrophages.	(137)
	Hydroxytyrosol	*Olea europaea* L.	Reduced the expression of the pro-inflammatory adhesion proteins ICAM1 and VCAM1 in HUVECs. Inhibited the pro-inflammatory cytokine (TNF-α) reduces the expression of COX-2 and promotes atherogenic processes. It regulates IL-1α, TNF-α, and ICAM-1, VCAM-1, and E-selectin mRNA synthesis.	(131, 213, 214)
	Probiotics, prebiotic and postbiotics	*Lactobacillus plantarum, Akkermansia muciniphila, Bifidobacterium lactis* Inulin, and fructans Short-chain fatty acids (SCFAs) such as butyrate	Gut probiotics have shown an immunomodulatory effect via upregulating Treg activity, suppress (Th) cells activity, alter the Th1/Th2 ratio, and influenced the subsets ratio of M1/M2 macrophages. Prebiotics diminished cholesterol levels and atherosclerotic lesions in mice. Regulated DCs, epithelial cells, Treg, effector lymphocytes, NK T cells, and B cells. Reduced the secretion of DC IL-12 and IL-6 cytokine and stimulated Treg cells through DC.	(130, 215, 216)
	Omega-3 PUFAs	Fish and other seafood	Reduced the expression of several important atherosclerotic markers such as IL-6, and TNF-α, in both murine and human macrophages. It also increased the expression of cholesterol efflux genes and decreased the expression of LDL-uptake genes.	(131, 217)
	Vitamin D	Fish, egg yolks, etc.	Attenuated the production of TNF-α and IL-1b and decreased expression of CD80 and CD86.	(145)
Diabetes	*Lagerstroemia speciosa*	Banaba leaf	Corosolic acid decreased the blood sugar levels in human subjects. Attenuated the differentiation of 3T3-L1 cells into adipocytes.	(148)
	Fenugreek	*Trigonella foenum-graecum*	Reduced the damage of β-cells in pancreatic islet of diabetic rats.	(150, 221)
	Bitter melon	*Momordica charantia*	The pancreas treated with bitter melon showed improved production of Langerhans islet cells. It suppressed blood glucose levels, prevented the intestine from absorbing the glucose, and improved pancreatic β-cells to produce insulin.	(219)
	α-Lipoic acid	Broccoli	Induced the differentiation of Th1 and Th17. Inhibited NF-κB activation induced by TNF. Suppressed IFN-γ and IL-4 produced by CD4+T.	(220)
	Dioscorea	*Dioscorea opposita*	Improved the TNF-α secretion by splenic lymphocytes, secreting phagocytosis, and aiding macrophages. Exerted anti-inflammatory effects on IL-6 leading to release of GLP-1 by intestinal L cells.	(221)
	*Allium sativum*	Alliaceae	Showed activity as α-amylase inhibitor, hypoglycemic, α-glucosidase inhibitor. Reduced the production of TNF-α, IL-6, IL-1β, and IFN-γ	(154, 222)
	Prebiotics and probiotics	*Lactobacillus plantarum Lactobacillus rhamnosus Lactococcus lactis Bifidobacterium animalis Lactobacillus reuteri*	Probiotics showed the potential to reduce the serum concentration of IL-6, TNF-α, and hs-CRP, which are the major risk factors for inflammation-dependent metabolic diseases like type-2 diabetes.	(223, 224)
Osteoporosis	Ashwagandha extract	*Withania somnifera*	Significantly increased immunomodulatory response at dose 60 mg in healthy adults (*p* < 0.05). Significantly increased the production of lymphocytes in randomized clinical study in test group. Increased production of IL-12, IFN-γ and subsequently decreased TGF-β, IL-10, and IL-4.	(163, 225)
	Guggul extract	*Commiphora*	Inhibited the activation of NF-κB by suppressing the levels of receptor activator of NF-κB.	(223)
	Arjuna	*Terminalia arjuna*	Downregulated the gene expression of pro-inflammatory cytokines in colitic rats	(230)
	Peptidoglycan	Mushroom	Increased the secretion of IL-12, IL-2, IL-6, IFN-γ and TNF-α	(228, 230)
	Probiotics	*Lactobacillus (L) reuteri, Bifidobacterium lactis L. paracasei*, and *L. plantarum*	Increased the production of CD4+T cells producing RANKL, IL-17, and TNF-α, thereby improving osteoclastogenesis	(231)
	Prebiotics	Inulin, fructooligosaccharides, and galactooligosaccharides	Decreased TNF-α and T cell expression along with greater osteoclast numbers.	(231)
Polycystic ovarian syndrome	Probiotics and prebiotics	*Lactobacillus casei, Bifidobacterium lactis* Oligosaccharides, inulin	Reduced the inflammatory cytokine-like TNF-α, IL-6, and IL-17a.	(112)
	Ashwagandha	*Withania somnifera*	Stimulated gonadotropin-releasing hormone and improved hormonal balance. Enhanced the level of IFN-γ, IL-2, and GM-CSF in mice. Increased the FSH and decreases LH, testosterone, and estradiol in letrozole-induced PCOS rats.	(232)
	Amla	*Emblica officinalis*	Inhibited cell proliferation and increased the production of IL-2 and IFN-γ production by lymphocytes.	(233)
	Apigenin	Parsley extract	Increased the production of progesterone and decreased estrogen and LH/FSH ratio. Decreased the follicle-stimulating hormone (FSH) and TOS. Decreased TNF-α, IL-6, and expression of NF-κB.	(234)
	Fenugreek extract	*Trigonella foenum-graecum* L.	It activated the CD4+ and CD8+T cells immune response significantly.	(150)
	Cinnamon bark extract	Cinnamon quills	Decreased systemic levels of IFN-γ, and it can inhibit anti-CD3 Ab-stimulated IFN-γ	(235)
	Vitamin D	Fish	Increased the production of IL-10, IL-1b, VEGF, and GM-CSF from NK cells and decreased the production of IFN-γ and TNF-α secretion from NK cells	(173, 174)
Non-alcoholic fatty liver diseases	Silymarin	*Silybum marianum*	Improved various lipid parameters (TC, C-LDL, HDL-C, and TG). Induced anti-inflammatory activity with reduction of transaminases levels. Decreased NF-κB signaling.	(178, 236)
	Oleic acid and linoleic acid	Ginseng seed oil	Reduced hepatic insulin resistance and enhanced expression of genes associated with β-oxidation. Decreased expression of adipogenic genes Srebf1 and Mlxip1.	(234)
	Alpha-lipoic acid	Broccoli	Decreased the production of cytokines IL-6 and increased serum adiponectin.	(238)
	Quercetin	Apples, grapes	Reduced the mitochondrial damage and SMAD2/3 signaling. Mitigated inflammation and oxidative stress suppressed TGF-β signaling to alleviate NAFLD.	(239)
	Probiotics	*Lactobacillus, Streptococcus, Lactococcus, Enterococcus, Bifidobacterium, Bacillus*, and *Clostridium.*	Inhibited inflammatory signaling, like JNK and NF-κB and restored the reduced hepatic cellular immunity caused by an HF diet.	(82)
	Vitamin-E	Citrus fruits	It decreased TNF-α, IL-2, IL-4, IL-6, and IL-8 and simultaneously increased the production of adiponectin. Its potent antioxidant activity caused a diminution of TGF-α and NADPH- oxidase TGF-β.	(240)
	Vitamin D	Fish, red meat, and egg yolks.	Prohibited the hepatic expression of pro-fibrotic mediators like PDGF and TGF-β. Significantly reduced CRP and TNF-α after consumption	(241)
Obesity	Probiotics and prebiotics	*Lactobacillus, Bifidobacterium, Saccharomyces, Streptococcus*, and *Enterococcus*,	Preserved intestinal permeability, reduced pro-inflammatory cytokines, protected intestinal barrier. Prebiotic-fed mice showed a low profile of several pro-inflammatory cytokines and diminished hepatic expression of inflammatory and oxidative stress markers.	(188)
	EGCG	Green tea	Decreased the production of pro-inflammatory cytokines like IL-2, IL-6, TNF-α, and IL-1β Raised the expression of Foxp3, IRF4, and IL-10, and impeded the expression of TLR4 TNF-α cytokines.	(242)
	Curcumin	*Curcuma longa*	Curcumin altered circulating concentrations of IL-1β, IL-4, and VEGF on 37 patients	(189)
	Arjunarishta	*Terminalia arjuna*	Significantly decreased gene expression of TNF-α and increased PGC-1α and IRS-1.	(243)
	Hydroxyl isoleucine	Fenugreek	Down-regulated a TNF-transforming catalyst which causes the change of mTNF to sTNF	(243)
	Allicin	*Allium sativum* L. (garlic)	Inhibited NO, TNF-α, and IL-1β by inhibiting NF-κB signaling pathway in LPS-stimulated J774A.1 macrophages	(244)
	Boswellic acid	Boswellia	Participated in the regulation of immune system through inflammation and autoreactive T cells. It showed anti-inflammatory and anti-obese immunomodulatory effects.	(246)
Aging	Curcumin	*Curcuma longa*	Inhibited NF-αB signaling-dependent inflammation and decreased the production of IL-8.	(247)
	Withaferin A	Ashwagandha	Responsible for most of the antiaging effects on the signaling pathways. Inhibited NF-κB activation by binding to the inhibitor (IKKβ) preventing phosphorylation of Iκβ	(248, 249)
	Prebiotics and probiotics	*Lactobacilli, Bifidobacteria*	Increased the production of IL-12 and NK cell activity.	(250)

### Atherosclerosis

Atherosclerosis is elucidated as an accumulation of cholesterol and conscript of macrophages into artery walls yielding plaques (130). As mentioned in the previous section, gut dysbiosis has been proposed to be directly associated with acute or chronic dysfunctions of atherosclerosis in the host. Intriguingly, in the last two decades, a number of nutraceuticals have shown their potential toward the management of atherosclerosis (131, 132). Much to our interest, preclinical and clinical evidences have been accumulated for their immunomodulatory role in the disease management as well. For instance, curcumin inhibited the production of IL-8, MIP-1α, MCP-1, IL-1β, and TNF-α by LPS-stimulated on human peripheral blood monocytes and alveolar macrophages. It ameliorates experimental autoimmune myocarditis (EAM) by reduced the inflammation in inflammatory macrophages and polarized M0 and M1 macrophage to M3 phenotype (133, 134). In addition, other groups also revealed that phytochemicals such as *Terminalia arjuna* extract had a prominent effect on the management of atherosclerosis. For example, in 2009, Halder et al. reported that T. arjuna possesses anti-inflammatory potential against some phlogistic agents as well as antinociceptive activity plausibly mediated via opioid receptors (135, 136). Similarly, Da Yeon Lee et al. (137), demonstrated that Allicin minimized inflammatory cytokines expression in murine such as IL-6, IL-1β, and TNF-α in macrophages stimulated with LPS (138). It was also demonstrated that hydroxytyrosol has played a major role toward diminishing cytokines IL-12 and IL-23 and Th1 and Th17 activation inhibiting atherosclerosis progression (139, 140). Similarly, in Qiang Wan et al. demonstrated that Berberine has decreased serum levels of IL-6 and TNF-α, which played a key role in pathogenesis of atherosclerosis (141). Interestingly, green tea extract increased the production of nitric oxide leading to enhanced vasodilation. Besides that, flavanols exhibited vasodilation plus lessened circulating oxLDL levels after 5 weeks (128). Moreover, prebiotics, probiotics, and postbiotics (SCFAs) are showing promising results toward the homeostasis of gut microbiota which is leading to counter the onset of atherosclerosis. For example, *Lactobacilli* have shown immunomodulatory effects against atherogenesis such as enhancement of the activity of Tregs, suppression of Th1, Th17, modification of Th1/Th2 ratio, influencing the subsets ratio of M1/M2 macrophages (142, 143). Few other groups reported that Mediterranean diet, antioxidant phytonutrient like coenzyme Q10, bioactive compounds like vitamins D, E, A and C, polyunsaturated fatty acids (ω-3 and ω-6), etc. are showed prominent effects for management of CVD (131, 144, 145). Taken together, immunomodulatory nutraceuticals can be used alone or concurrent with other pharmaceutical treatment modalities to prevent and manage atherosclerosis and to ameliorate the QoL of the patients.

### Diabetes

Diabetes mellitus (DM) is a metabolic disease, which takes its origin from several genetic and environmental factors and characterized by insulin resistance or impaired production of insulin hormone from pancreatic β-cell, which creates an enormous health burden with micro as well as macrovascular complications. As reported by International Diabetes Federation in 2019, diabetic population was estimated to be 463 million and it was predicted to increase up to 700 million by 2045 (146). As mentioned in the previous section, alteration in gut microbial composition can largely contribute to the onset of the disease. Herein, we aim to summarize some immunomodulatory nutraceuticals including botanicals and probiotics, which can help in the management of diabetes. In 2012, Toshihiro Miura et al. reported that *Lagerstroemia speciosa* L., a rich source of corosolic acid as well as ellagitannins, was responsible for blocking the activation of NF-κB in a dose- and time-dependent manner in H9c2 cell line, which modulated anti-inflammatory action resulting in inhibition of diabetes-induced cardiomyocyte hypertrophy (147, 148). Similarly, other research groups (Sneha J. Anarthe et al. and Neelam Makare et al.) demonstrated that 500 mg/kg of Trigonella foenum-graecum increased the population of lymphocytes and T cell (149–151). Bitter melon, α-lipoic acid, dioscorea, allium sativum, and amaranthus have also shown positive antidiabetic immunomodulatory effects (152–156). In Bahare Salehi et al. reported that α-lipoic acid has the potential to be used for the management of diabetes and other NCDs including Alzheimer (157). In addition, Giuseppe Derosa et al. recommended that L-carnitine, α-lipoic acid, berberine, and ω-3 fatty acids might be useful toward the management of diabetes (158). Probiotics (*bifidobacteria, lactobacilli*, and *Streptococcus thermophilus*) have been demonstrated to be a powerful arsenal to combat central components of metabolic syndrome, like T2D (159). The detailed immunomodulatory effects of the nutraceuticals toward the inhibition of diabetes are summarized in Table 2 and Figure 5.

### Osteoporosis

Osteoporosis, mostly afflicting postmenopausal women, is a bone metabolic disorder characterized by bone loss leading to an enhanced risk of fracture (110). As reported by Jing Yan et al. in resident microbes promote bone formation and prolonged exposure results in net skeletal growth. Hormone IGF-1, produced by microbiota, promotes bone development and remodeling (160). Other groups also demonstrated that gut microbiota played a key role in bone homeostasis, regulated bone metabolism through various pathways, endocrine system and through immune system, and promote on calcium balance (110, 161). Importantly, with a continuous accumulation of knowledge, it was disclosed that the “brain–gut” axis may be a potential target for the bone, which affects the onset and propagation of osteoporosis. Yuan-Wei Zhang et al. demonstrated that the monitoring of TNF^+^ T and Th17 inflammatory cells in the bone marrow improved the overall inflammatory state. It may be called the “brain-gut–bone” axis (162). Accumulated preclinical evidence also account for the nutraceuticals playing a potential role in the management of bone loss past menopause. Some phyto-nutraceutical compound showed positive results against osteoporosis. For instance, in 2021, Tharakan and co-workers reported a positive immunomodulatory effect of *Withania somnifera* extract during preclinical studies which significantly increased the production of cytokines IFN-γ, IL-4, and CD45+, CD3+, CD4+, CD8+, and CD19+ NK cells (163). Moreover, Zaffar Azam et al. recently reported that guggul extract, arjuna, coriolus versicolor, and Punica granatum showed a promising immunomodulatory effect for the management of bone health (164). Similarly (165, 166) reported that probiotics and prebiotics can help to preserve gut barrier integrity, protect against pathogenic microorganisms, and promote alteration of CD4+ T cell activation, which can modulate osteoclastogenic cytokine production (165, 166). A research group demonstrated that vitamin D has played some role in the prevention and management of osteoporosis via regulating calcium–phosphorus homeostasis controlling Treg differentiation, reducing Th17 cell response, and inflammatory cytokines secretion (167, 168). The details immunomodulatory effect is depicted in below Figure 5 and Table 2.

### Polycystic ovarian syndrome

Polycystic ovarian syndrome (PCOS) is a gynecologic endocrine metabolic disease, particularly affecting women of reproductive age (111). As evident from prior scientific research, gut microbiota can influence the pathogenesis and clinical manifestations of PCOS (84). Recently, Fang-fang He et al. reported that gut dysbiosis occurs in PCOS animal models and patients with PCOS which hint at an apparently ambiguous role in the prevalence of *Prevotellaceae* (169). To our intrigue, other nutraceutical compounds have also shown their potential toward the management of PCOS. For example, probiotics and prebiotics (*Lactobacillus casei, Bifidobacterium lactis, Lactobacillus plantarum, Lactobacillus rhamnosus, oligosaccharides, inulin*) have shown a reduction in inflammatory cytokines (TNF-α and IL-17a) in patients with PCOS as reported by Gamze Yurtdaş et al. (111, 170). Similarly, other research groups (Bilal Bin-Hafeez et al. and Sneha J. Anarthe et al.) demonstrated a dose-dependent immunomodulatory activity of methanolic extract of fenugreek (149, 150). It has also been demonstrated that phytonutrients such as ashwagandha, amla, apigenin, and cinnamon bark extract furnish promising immunomodulatory effects for the management of PCOS, the details are summarized in Table 2 (171, 172). Moreover, in Kuniaki Ota et al. demonstrated that vitamin D has shown an immunomodulatory effect inhibiting the proliferation of Th1 cells and limit their cytokine production, such as IFN-γ, IL-2, and TNF-α. Some contradictory behavior of vitamin D has also been recorded (173, 174). In summary, immunomodulatory nutritional intervention can be used alone to prevent and manage PCOS or concurrent with other pharmaceutical treatment modalities to ameliorate the quality of life of the patients.

### Non-alcoholic fatty liver disease

Non-alcoholic fatty liver disease (NAFLD) is one of the leading causes of mortality and morbidity all over the world. The prevalence of NAFLD is projected in 2020 to increase up to 56% in the next 10 years (175). It is majorly caused by an accretion of fatty acid content greater than 5% of liver weight (176). Moreover, gut microbiome, its metabolites and their interactions with the immune system - together entails the pathogenesis of NAFLD and hepatocellular carcinoma (HCC) via gut–liver axis (177). Herein, we highlight some nutraceuticals, vitamins, prebiotics, and probiotic supplements, which showed positive outcome in the management of NAFLD in few preclinical and clinical settings. For instance, in Annalisa Curcio et al. demonstrated that Silybum marianum, having antioxidant and anti-inflammatory activity and comprising ∼70–80% of silymarin with a mixture of flavonolignans and silybin, improved steatosis and liver enzymes with patients with NAFLD (178). Similarly, other research groups showed that gut microbiota–derived tryptophan metabolites (I3A) weakened the expression of TNF-α, IL-1β, and MCP-1 on macrophages exposed to palmitate and lipopolysaccharide (179). In addition, probiotics/synbiotics are used as supportive supplement diets to reduce inflammation, hepatic steatosis, and liver stiffness as shown in meta-analysis (180, 181). Importantly, in 2017, Kelishadi and colleagues demonstrated that a probiotic blend containing *Bifidobacterium lactis* (DSMZ 32269), *Lactobacillus acidophilus* (ATCC B3208), *Lactobacillus rhamnosus* (DSMZ 21690), and *Bifidobacterium bifidum* (ATCC SD6576) had shown a positive result after 12 weeks of treatment on patients with pediatric NAFLD along with reduced liver injury compared to placebo treatment (180–182). Immunomodulatory effects of various nutraceuticals for the management of NAFLD are summarized in below Table 2.

### Obesity

Though obesity cannot strictly be stated as NCD, it is a well-known risk factor that can beget several metabolic disorders such as CVD and T2DM. It is proven that certain gut bacterial genera are associated with obesity, making microbiome modulation an attractive tool for its management. In Aline Corado Gomes et al. demonstrated various molecular patterns that lead together to obesity, such as immune system, lipid metabolism, satiety hormones, nutrient metabolism, and microbiota–adipose tissue axis (183). Thus, maintaining homeostasis and attenuation of the exaggerated inflammatory reaction owing to dysbiosis is required in order to prohibit the onset of obesity which makes probiotics a therapeutic modality against the disorder. For example, *Lactobacillus gasseri SBT2055, Bifidobacterium L66*, and *Bifidobacterium adolescentis* drew attention as they altered the composition of gut microbiota and affected food intake, appetite, body weight, body composition, and metabolic functions involving GI pathways (184). A few other groups also evaluated the effects of prebiotics on obesity. For instance, prebiotic-fed mice showed a low profile of several pro-inflammatory cytokines and diminished hepatic expression of inflammatory and oxidative stress markers that could maintain gut homeostasis and control obesity (185–188). Phytonutrients have also shown some promise in obesity management. As reported by Shiva Ganjali et al. in (162), curcumin showed a promising role in obesity management. When obese individuals treated with 1 g curcumin/day in a 4-week long randomized crossover trial, the mean serum IL-1β (*p* = 0.042), IL-4 (*p* = 0.008), and VEGF (*p* = 0.01) were found to be significantly reduced (189). Similarly, other authors recently reported that phytonutrients such as green tee, arjunarishta, fenugreek, and boswellic acid played a major role toward controlling obesity (190, 191). Table 2 elucidates the immunomodulatory effects of these ingredients.

### Aging

Aging is a complex phenomenon spawned out of the interaction of environmental, genetic, and/or epigenetic events interfering with body’s functions with time. Age-related chronic disorders are progressively increasing due to the increased life expectancy in the elderly population. These can be attributed to the compositional shift in the gut microbiota which remains associated with low-grade inflammation and innate immunity activation which can trigger many metabolic dysfunctions. Therefore, gut microbiota can be considered as a target for the elderly population to reverse aging as well as inhibit metabolic ailments (117). Here, we mention the effects of some phytonutrients, prebiotics, and postbiotics supplements which have demonstrated their potential to ameliorate the gut dysbiosis and to direct toward healthy aging (192). In preclinical studies, curcumin demonstrated symptomatic reduction in some age-related diseases such as CVD, T2DM, and cancer owing to its well-known anti-inflammatory property via inhibiting NF-αB signaling (193). Other research groups have also demonstrated that Shilajit, Withaferin A, and prebiotics and probiotics can play crucial roles in the management and prevention of aging-associated immune compromise and metabolic diseases including cancer, diabetes, and obesity (194, 195). Probiotic supplementation increased NK cell and phagocytic activity, mostly effective in elderly population. It also ameliorated the detrimental effects of malnutrition on immunity in elderly people by improving their nutritional and immune status, as demonstrated by increasing levels of serum albumin and intestinal immunoglobulin A (IgA) production (196). In 2021, Xin Fang et al. demonstrated that the antiaging effects of the probiotic has been regulated intestinal microbiota and inhibited TLR4/NFκB-induced inflammation in mouse model (197). The effect of a probiotic blend of two *Bifidobacterium* species in a South Korean elderly (65 years) population after 12 weeks was measured to have reduced abundance of *Prevotellaceae* family and *Eubacterium*, *Clostridiales*, and *Allisonela* order which results in improved cognitive function and stress management capacity further reinforcing the significance of gut–brain axis. The details of immunomodulatory effects of various nutraceuticals on aging are depicted in Table 2.

Summarizing the above facts, it is quite palpable to decipher the mechanistic roles of the vast majority of the nutraceuticals are playing, which can be validated and translated into beneficial formulations with better efficacy.

## Future perspective

So far, the causative roles of microbes in non-communicable diseases, some cancers, and immunomodulation via nutraceutical management are recognizable. The prevalence of microbes in gut is dependent on several factors such as immunity, food, and external environment. Several other agents such as antibiotics, mutagens, or carcinogens can influence human microbiome via immunosuppression, oxygen deprivation, biofilm formation, etc. Though fecal microbiome transplant (FMT) technology from a healthy donor has gained popularity, the United States Food and Drug Administration (FDA) recently issued several restrictions on FMT and its trials after numerous infections and one death was reported (251–254). It is also important to study cohort for the health status, effect of food habits, and age of a person to harvest gut microbiome/fecal microbiome to get rid of accidental pathogenesis. Microbial biobanks can be established in various countries abiding by the law of the lands by collecting microbial communities from healthy donors at various stages of their lives. Considering the degree and frequency of the NCD occurrence, these preserved microbial communities can be transplanted. Periodical preservation of fecal microbiome and/or gut microbiota can be a probable substitute in order to develop a personalized FMT for dysbiosis but deciding timeline for harvesting fecal microbiome for preservation is the biggest challenge. Metagenomic analysis methods can guide both taxonomic and functional information from diverse microbial groups. Despite significant efforts directed toward culturing and classifying microbial diversity within gut ecosystem, it still remains difficult to identify biological role of some microbial community that are low in number or nutrient provided for culturing is insufficient. Correct understanding of this can be exploited further to formulate immunomodulatory nutraceutical products for the management of several non-communicable diseases affected by dysbiosis of human microbiome. Another important aspect that should be exhaustively explored in the future is the prediction of the occurrence of NCDs and some cancers by analyzing the gut microbiome or fecal microbiome. Considering varying demography, food habits, and ethnicity, different cohorts can be chosen for experimentation and analysis. Once precisely predicted, numerous preventive measures can be taken to avoid the incidence of the maladies. Personalized precision nutraceutical interventions can be suggested once the disease has been detected. Various nano-theranostics (therapy plus diagnostics) modalities can be discovered and utilized toward successful diagnosis and eradication of the disease. For effective execution of these kind of studies, close collaborations between academic institutes, hospitals, and industries are required. More of these collaborations should be encouraged and implemented by the regulatory agencies.

## Conclusion

In summary, identification of the various microbes causing low-grade inflammation over a period of time in the human system which eventually leads to the onset of the pathogenesis of several NCDs and some cancers are of immense importance. It is also equally important to harness therapeutic benefits from the nutraceuticals in terms of their immunomodulatory activities toward inhibiting the pathogenesis and progression of the aforementioned maladies. Here, we attempted to present a broad summary of these two interconnected phenomena which can open new avenues to address the bleak ramifications of various NCDs and cancers. We envision that a plethora of novel therapeutics can be generated based on systematic analysis of the cause and effectuating the personalized precision medicine which can be begotten from the immunomodulatory nutraceuticals in recent future.

## Author contributions

AM, KN, and RC contributed to the conceptualization. AK, KS, DB, KN, RC, PP, and AM contributed to the manuscript writing. AM contributed to the editing. All authors contributed to the article and approved the submitted version.
